# Identifying Fibroblast Growth Factor Receptor 3 as a Mediator of Periosteal Osteochondral Differentiation through the Construction of microRNA-Based Interaction Networks

**DOI:** 10.3390/biology12111381

**Published:** 2023-10-28

**Authors:** Leah M. Wells, Helen C. Roberts, Frank P. Luyten, Scott J. Roberts

**Affiliations:** 1 Department of Comparative Biomedical Sciences, The Royal Veterinary College, London NW1 0TU, UK; lwells@rvc.ac.uk; 2 Department of Natural Sciences, Middlesex University, London NW4 4BT, UK; h.roberts@mdx.ac.uk; 3 Skeletal Biology and Engineering Research Centre (SBE), KU Leuven, 3000 Leuven, Belgium; frank.luyten@kuleuven.be

**Keywords:** microRNA, osteochondral differentiation, fracture repair, osteoarthritis

## Abstract

**Simple Summary:**

The cartilage-to-bone transition is an essential process in healthy bone development and repair. Our previous work has shown that when the cells found within the human periosteum (the membrane surrounding the bone) are cultured in human serum (HS) as opposed to the standard animal serum (FBS), these cells have greater bone-forming capacity as assessed in an ectopic assay in nude mice. What is not understood is the molecular interactions that permitted this enhanced biological potency. Herein, virtual networks are created to identify the key proteins driving increased bone formation from these cells. Key signalling factors were identified through a network analysis, where FGFR3 was pinpointed as a major differential regulator between cells grown in HS and cells grown in FBS. This analysis was validated through an analysis of human-derived periosteal progenitor cells (PDCs) containing a constitutively active (ca) FGFR3. Following removal and analysis, we found that the FGFR3-ca cells that were implanted on bone void filler scaffolds in mice had an abundance of bone and cartilage that were present compared to the scaffold containing normal/healthy cells. This suggests that these cells were undergoing enhanced cartilage-to-bone transitions and that this protein may be a potentially novel therapeutic target for diseases where the cartilage-to-bone transition is affected such as during poor fracture healing.

**Abstract:**

Human periosteum-derived progenitor cells (hPDCs) have the ability to differentiate towards both the chondrogenic and osteogenic lineages. This coordinated and complex osteochondrogenic differentiation process permits endochondral ossification and is essential in bone development and repair. We have previously shown that humanised cultures of hPDCs enhance their osteochondrogenic potentials in vitro and in vivo; however, the underlying mechanisms are largely unknown. This study aimed to identify novel regulators of hPDC osteochondrogenic differentiation through the construction of miRNA-mRNA regulatory networks derived from hPDCs cultured in human serum or foetal bovine serum as an alternative in silico strategy to serum characterisation. Sixteen differentially expressed miRNAs (DEMis) were identified in the humanised culture. In silico analysis of the DEMis with TargetScan allowed for the identification of 1503 potential miRNA target genes. Upon comparison with a paired RNAseq dataset, a 4.5% overlap was observed (122 genes). A protein–protein interaction network created with STRING interestingly identified FGFR3 as a key network node, which was further predicted using multiple pathway analyses. Functional analysis revealed that hPDCs with the activating mutation FGFR3^N540K^ displayed increased expressions of chondrogenic gene markers when cultured under chondrogenic conditions in vitro and displayed enhanced endochondral bone formation in vivo. A further histological analysis uncovered known downstream mediators involved in FGFR3 signalling and endochondral ossification to be upregulated in hPDC FGFR3^N540K^-seeded implants. This combinational approach of miRNA-mRNA-protein network analysis with in vitro and in vivo characterisation has permitted the identification of FGFR3 as a novel mediator of hPDC biology. Furthermore, this miRNA-based workflow may also allow for the identification of drug targets, which may be of relevance in instances of delayed fracture repair.

## 1. Introduction

The periosteum is a thin membranous tissue that encapsulates bone and consists of two distinct layers. The outer fibrous layer is collagenous, with fibroblasts found intertwined with collagen fibrils. Underneath is the inner cambium layer, which is in contact with the bone surface and is rich in skeletal progenitor cells, which are termed periosteum-derived progenitor cells (PDCs) upon isolation and culture. These layers are highly vascularised and innervated, providing the bone with a blood supply and sensation [1,2].

**Figure 1:**
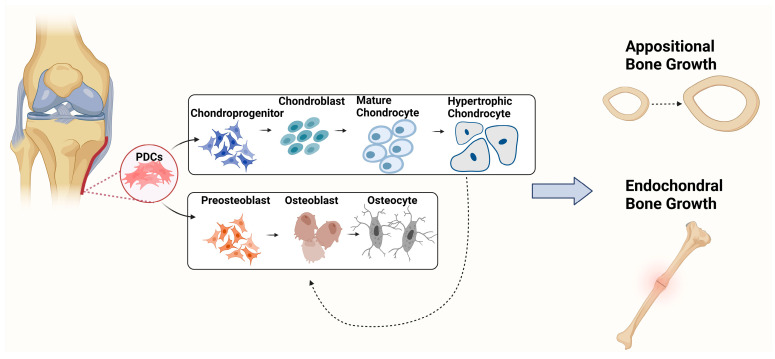
Periosteal osteochondral differentiation. Progenitor cells reside in the membrane surrounding the bone (termed the periosteum). These cells have both chondrogenic and osteogenic differentiation potentials, permitting appositional and endochondral bone growth. Created with BioRender.com.

PDCs have chondrogenic and osteogenic differentiation potential and have been shown to have roles in appositional bone growth and endochondral bone formation during fracture repair (Figure 1) [3,4]. However, it is not yet known whether these cells have dual differentiation capabilities or whether these are two separate progenitor populations [5]. During appositional bone growth, endosteum-derived osteoclasts resorb the internal surface of the bone surrounding the medullary cavity [6]. Simultaneously, periosteum-derived osteoblasts deposit and form new bone on the outer surface, resulting in an increased bone diameter [7]. Furthermore, PDCs have chondrogenic capabilities and play important roles in providing progenitor cells for the endochondral ossification healing process during fracture repair, particularly in non-stabilised fractures [8]. Following bone injury, PDCs invade the fracture site and undergo chondrogenic differentiation, resulting in the formation of a cartilaginous callus intermediate known as the “soft callus”. Mature chondrocytes continue to differentiate to hypertrophy, which initiates the mineralisation and degradation of the soft callus. This terminates with chondrocyte apoptosis or chondrocyte transdifferentiation to osteoblasts, followed by hard callus formation, which is subsequently remodelled into woven and lamellar bone via osteoblast and osteoclast activities over time [9].

Notably, the PDC number and chondrogenic potential have been shown to markedly reduce with age, resulting in poorer regenerative capabilities of the bone [10,11]. A delay or failure of the endochondral ossification process occurs in up to 9% of fractures, which has a significant impact on a patient’s quality of life due to invasive reparative surgeries and fracture site pain [12]. Additionally, aberrant bone formation in osteoarthritis (OA) (as observed during osteophyte development) is thought to be driven by periosteum-derived progenitor cell aberrant differentiation [13].

MicroRNAs (miRNAs) play essential roles in the post translational control of gene expression through the direct degradation of mRNA or the inhibition of translation via the formation of the silencing complex [14]. miRNAs have the ability to bind and silence multiple transcripts [15,16]. Indeed, it has been predicted that all mature mRNAs contain miRNA binding sites, indicating the importance of miRNA–mRNA interactions in ensuring the efficient control of gene expression [17]. Defining the dynamics of these miRNA–mRNA interactions during periosteal osteochondral differentiation will offer novel insights into the key drivers of this complex differentiation process. Furthermore, network identification may reveal potentially novel therapeutic targets for conditions characterised by aberrant osteochondral differentiation, such as fracture non-union and OA.

Previous work by our group has shown that humanised culture improves the translational potential of hPDCs through enhanced chondrogenic and osteogenic differentiation. Moreover, when these cells were implanted ectopically in vivo, these humanised cultured cells had greater bone-forming capabilities [18]. However, the regulatory networks that underpin this enhanced potency are unknown. This study aims to construct a microRNA-mRNA-protein regulatory network that underpins novel processes that may be involved in hPDC osteochondrogenic differentiation through the identification of key hub genes such as FGFR3 without the need for serum characterisation. Additionally, we suggest a novel understanding of the role of FGFR3 in human periosteal cells, which may be of particular therapeutic relevance for fracture repair. 

## 2. Materials and Methods

### 2.1. hPDC Isolation and Culture

hPDCs were isolated from biopsies obtained from patients undergoing orthopaedic surgery [19]. Human Medical Research (KU Leuven) approved all procedures, and patient informed consent was obtained. Subsequently, hPDCs (6 donors, 18.6 ± 8.9 years old, pooled, *n* = 3) were expanded in growth media (high-glucose DMEM (Gibco), 10% batch-tested FBSand antibiotic-antimycotic solution (100 units/mL penicillin, 100 μg/mL streptomycin and 0.25 μg/mL amphotericin B); Invitrogen) until passage 5. 

### 2.2. hPDC RNAseq and miRNA Analysis

To define the effects observed in our previous work with hPDCs in a humanised culture system [18], hPDCs were seeded at 1000 cells/cm^2^ and cultured in growth media containing 10% FBS (*n* = 3 Gibco batches) or human serum (47 donors, pooled, *n* = 3) for 6 days before processing for RNA, as described in Al Hosni et al. [20]. Additional miRNA analysis was also conducted. Briefly, RNA was extracted using the Illumina TruSeq Standard Total RNA Sample Prep Kit (Illumina, San Diego, CA, USA). RNA integrity was validated using a BioAnalyzer (Agilent, Santa Clara, CA, USA). RNAseq was performed at the Nucleomics Core (KU Leuven, Leuven, Belgium). Libraries were generated from 2 μg RNA using the TruSeq library prep kit (Illumina) as per the manufacturer’s recommendations, and sequencing was carried out on the HiSeq2000 (Illumina) with read lengths of 50 base pairs. Between 25.0 and 39.3 million reads were sequenced for each sample. All parameters and subsequent analyses are described in detail elsewhere [19]. To select genes, the corrected *p* value was set at <0.05, which resulted in 1319 differentially expressed genes (DEGs) (≥2-fold).

miRNA analysis was carried out via the nCounter system (Nucleomics Core, KULeuven), which utilises a chip system comprising 800 human miRNA probes, including 6 positive (a range of samples with known concentrations) and 6 negative controls, alongside 5 housekeepers. A normalisation factor was generated by calculating the median value from the top 100 miRNAs, which was then divided by the average counts of the top 100 miRNAs in the assay; each count was then transformed using this normalisation factor. miRNA counts with a differential expression of ≥Log_2_ and *p* ≤ 0.05 between the control (FBS cultured cells) and humanised culture (human serum cultured cells) were considered differentially expressed miRNAs (DEMis). This returned a list of 16 DEMis. 

### 2.3. Identifying High-Confidence Differentially Expressed miRNA-Regulated Genes

Target scan is an online database that uses the context++ model to predict miRNA target genes [21]. To identify gene targets of identified DEMis, each DEMi was searched on the Target scan database (www.targetscan.org; accessed on 10 March 2022), and gene targets were compiled, identifying 1503 potential target genes (with the exception of has-miR-720, which was not found within the target scan database). DEMi target genes were then cross-referenced with DEGs from the paired RNAseq dataset, and overlaps between the two datasets were identified using Venny2.0. This overlap dataset consisting of 122 high-confidence target genes was used for all downstream pathway analyses. 

### 2.4. PPI Network Construction

The 122 high-confidence target genes were input into the STRING online platform (www.string-db.org; accessed on 24 March 2022), which carries out functional enrichment analysis to generate protein–protein interaction (PPI) networks within a dataset. Analysis settings were set to display interactions with a minimum confidence score of 0.400 (medium) with a full STRING network type, the network edges were evidence of an interaction and line colours indicate the type of interaction evidence. All interaction sources were selected, and disconnected nodes were removed. 

### 2.5. Pathway Enrichment Analysis

High-confidence target genes that were previously identified were applied onto the PANTHER (www.pantherdb.org; accessed on 25 March 2022) online analysis platform via functional classification and the ShinyGO.0.77 (ShinyGO 0.77 (www.sdstate.edu) accessed on 25 March 2022) platform to identify pathways enriched within the gene dataset. For ShinyGo0.77, the analysis parameters were set to human species in the curated Reactome pathway database with an FDR cut-off of 0.05 and a pathway size minimum of 2 with the redundancy removed. 

### 2.6. hPDC-FGFR3(ca) In Vitro Cell Quantification via DNA Analysis

hPDCs were isolated from donors (hPDC (ca) donors: N540K N = 1, 5-year-old male; G380R N = 1, 5-year-old male; control donors: N = 2, control 1 = 3-year-old female; control 2 = 8-year-old male) as previously described. Cells were cultured in growth media (DMEM high glucose, 10% FBS, 1% antibiotic/antimycotic solution) until passage 5 [18]. Cells were seeded at 4.5 × 10^3^ cells/cm^3^ and cultured in growth media for 14 days before being processed for DNA quantification, as described by Chen et al. 2012 [22]. Statistical analysis was determined via one-way ANOVA and Tukey’s multiple comparison test; *n* = 3.

### 2.7. hPDC-FGFR3(ca) In Vitro Gene Expression Analysis of Chondrogenic Markers

Isolated hPDCs with and without FGFR3(ca) were cultured in growth media (DMEM high glucose, 10% FBS, 1% antibiotic/antimycotic solution) until passage 5. Chondrogenic differentiation of control and FGFR3(ca) hPDCs were assessed by culturing the cells in high-density micromasses (4 × 10^5^ cells per 10 μL) in the presence of chondrogenic medium (DMEM Nutrient Mixture F-12, 2% FBS, 1× insulin-transferrin-selenium-positive supplement, and 10 ng/mL transforming growth factor β1) for 6 days, and undifferentiated controls were cultured in growth media only. For osteogenic analysis, 4.5 × 10^3^ cells/cm^3^ were seeded for 48 h in growth media before culturing for 14 days in osteogenic medium (DMEM supplemented with 10% FBS, 100 nM dexamethasone, 50 μg/mL ascorbic acid, and 10 mM β-glycerophosphate) or standard growth media controls. RNA extraction was performed using the RNeasy Kit (Qiagen) according to the manufacturer’s instructions, and cDNA was generated from RNA via reverse transcription using Superscript III; Invitrogen. Quantitative real-time SYBR Green (Invitrogen) PCR was performed according to the manufacturer’s protocol, with mRNA levels normalised to hypoxanthine phosphoribosyltransferase 1 (HPRT1) expression, and relative gene expression was calculated using the 2-ΔCT method. Primer sequences included the following: HPRT1 F’: TGAGGATTTGGAAAGGGTGT, HPRT1 R’: GAGCACACAGAGGGCTACAA, FGFR3 F’: GTGACAGACGCTCCATCCTC, FGFR3 R’: CCAGCAGCTTCTTGTCCATC, SOX9 F’: TGGAGACTTCTGAACGAGAGC, and SOX9 R’: CGTTCTTCACCGACTTCCTC. Statistical analysis was carried out via two-way ANOVA and Tukey’s multiple comparison test; *n* = 3.

### 2.8. In Vivo Analysis of hPDC-FGFR3(ca) Osteochondral Differentiation

To determine whether hPDC-FGFR3(ca) cells had enhanced endochondral ossification capabilities, 1 × 10^6^ hPDCs or hPDC-FGFR3(ca) were seeded onto 21 mm^3^ cylindrical CopiOs™ bone void filler scaffolds composed of a calcium phosphate and collagen network or Collagraft™ composed of a hydroxyapatite–collagen hybrid matrix. Cells were allowed to adhere overnight at 37 °C with 5% CO_2_ in a humidified environment. NMRI-nu/nu mice were anaesthetised, and scaffolds were then implanted subcutaneously in the back cervical region before the wound was closed with surgical staples. Scaffolds remained implanted for 8 weeks before removal and processing for analysis. Briefly, extracted implants (*n* = 4/group) were fixed in 4% paraformaldehyde for 48 h. To visualise bone formation, micro-CT (µCT) imaging was carried out using the Skyscan 1172 system (Skyscan NV) with X-ray settings of 60 kV and 167 µA, using an aluminium 0.5 mm filter and a pixel size of 4.5 µm, and images were reconstructed using NRecon (Skyscan). Post imaging, scaffolds were decalcified in 20% EDTA/PBS (pH 7.5) solution for 2 weeks before being paraffin-embedded and processed for histological analysis via Toludine blue staining, as previously described [23]. For immunohistochemical analysis of phosphorylated Nuclear Factor kappa B (pNFκB), phosphorylated CCAAT/Enhancer-Binding Protein Beta (pC/EBPβ), and Bone Morphogenetic Protein (BMP7), sections were deparaffinised in histoclear and methanol. Antigen retrieval was performed via incubation with 10 mM sodium citrate (pH 6) at room temperature for 120 min before quenching for 10 min in 3% H_2_O_2_. Sections were washed in 0.1% Tween, TBS (TBST) and blocked for 30 min at room temperature in 20% donkey (BMP7) or goat (pNFκB; pC/EBPβ) serum/TBST (blocking buffer). Primary antibodies were diluted in blocking buffer (pNFκB 1:100, AbCam; pC/EBPβ 1:100, AbCam; anti-BMP7 10 µg/mL, Pfizer) and incubated overnight at 4 °C. For pNFκB and pC/EBPβ analysis, the sections were probed using the ABC kit (VectaStain anti-rabbit) as per the manufacturer’s instructions. BMP7 sections were probed with HRP-Anti-Chicken (1/100) for 30 min. All staining was revealed using DAB+/Chromogen and counter stained with haematoxylin before mounting. All procedures were approved by the local ethical committee for animal research (KU Leuven), and animals were housed according to the guidelines of the Animalium Leuven (Katholieke Universiteit Leuven).

## 3. Results

**Figure 2:**
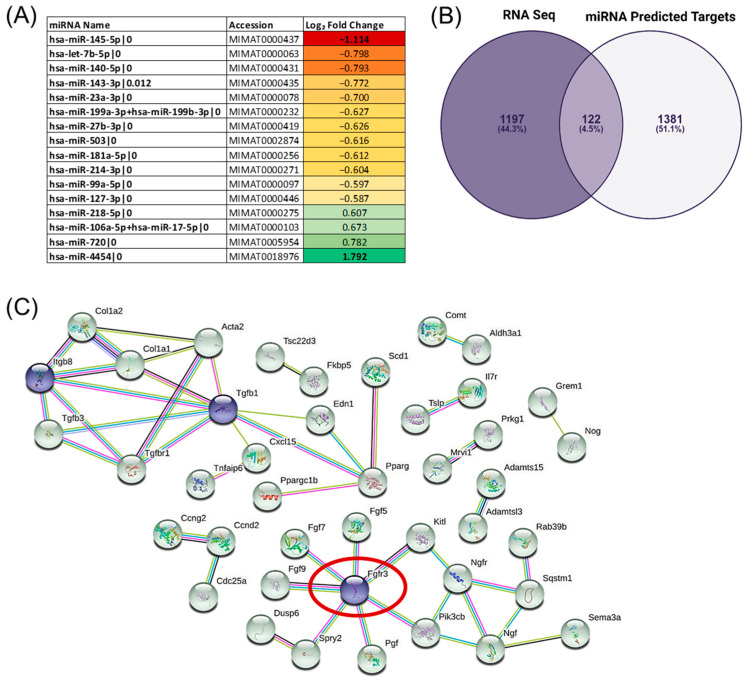
miRNA-RNA regulatory network analysis reveals FGFR3 as a hub gene. (**A**) Table highlighting the top up- and downregulated miRNAs (DEMis) from 16 differentially expressed miRNAs in the humanised condition compared to FBS with their respective Log_2_ fold change. Colour scale represents Log_2_ fold change, with red being the top downregulated and green the top upregulated (**B**) Venn diagram displaying the 4.5% overlap (122 genes) from the miRNA predicted targets as identified via TargetScan (1503 genes) with paired RNAseq data (1319 genes). These 122 genes were identified as high-confidence gene targets. Produced using Venny 2.0 [25]. (**C**) STRING analysis of the 122 high-confidence target genes identified FGFR3 as a key network node (circled in red) in addition to other potential network nodes including ITGβ8 and TGFβ1 (highlighted as purple nodes).

It is well established that the presence of miRNAs does not necessarily relate to functional activity [24]. It is therefore important to ensure that the network analysis of the predicted miRNA target genes is as stringent as possible. As highlighted in Figure 2, 16 differentially expressed miRNAs (DEMis) were identified via the human nCounter system, with hsa-miR-145-5p being the top downregulated DEMi (−1.11 Log_2_ fold change (FC) in humanised culture vs. FBS) and hsa-miR-4454 being the top upregulated DEMi (+1.79 Log_2_ FC in humanised culture vs. FBS).

To identify high-confidence gene targets, the TargetScan dataset generated from the 16 differentially expressed miRNAs (1503 DEMis) was cross-referenced with the paired RNAseq data (≥2-fold; *p* ≤ 0.05; 1319 DEGs), revealing 122 high-confidence target genes. These genes were analysed via STRING to identify the protein–protein interaction networks, which permitted the identification of Fibroblast Growth Factor Receptor 3 (FGFR3) as a key network node, which displayed a direct interaction with seven other proteins via >3 connections per interaction, suggesting that these interactions are of high confidence. Integrin Subunit Beta 8 (ITGβ8) and transforming growth factor beta 1 (TGFβ1) were also identified as potential network nodes displaying five and four direct interactions with >3 connections per interaction, respectively.

**Figure 3:**
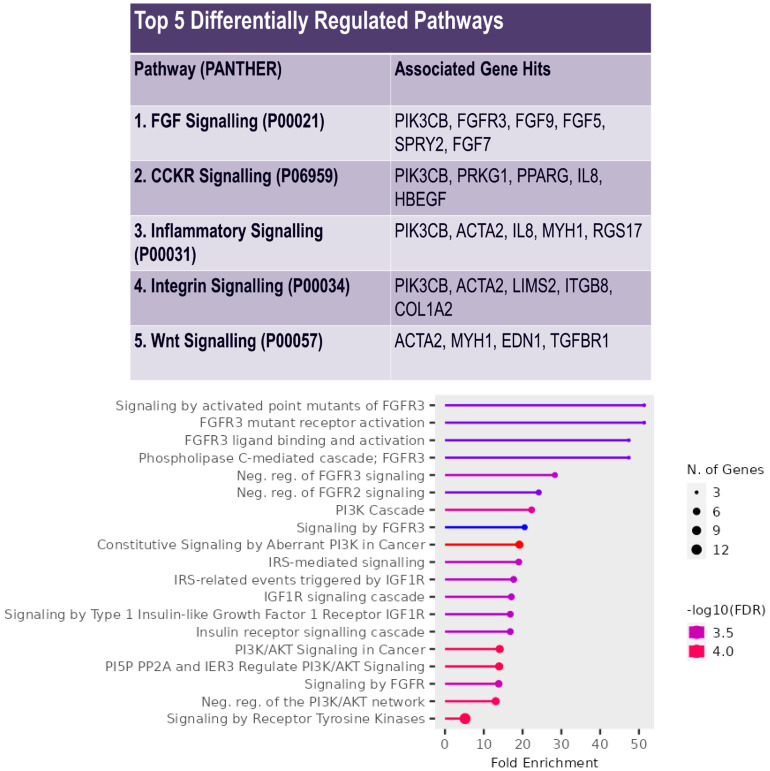
FGFR3 signalling identified as a major differentially regulated pathway via pathway analysis. (**Top**) Table highlighting the top 5 differentially regulated pathways in the 122-gene dataset according to PANTHER. FGF signalling identified as the top differentially regulated pathway with 6 associated gene hits. Other differentially regulated pathways include CCKR signalling, inflammatory signalling, integrin signalling, and Wnt signalling. (**Bottom**) Reactome enrichment analysis via ShinyGo v0.77 displays FGFR3 mediated pathways to be significantly enriched by up to 50-fold within the dataset.

Further network interrogations via PANTHER and ShinyGo allowed for a pathway enrichment analysis (Figure 3). The PANTHER analysis on the 122-gene dataset revealed fibroblast growth factor (FGF) signalling to be the top differentially regulated pathway with six associated gene hits including FGFR3. This was closely followed by cholecystokinin receptor (CCKR) signalling, inflammatory signalling, and integrin signalling, all with five associated gene hits, and Wnt signalling, with four associated gene hits. An alternative pathway analysis of the Reactome pathways via ShinyGo identified the majority of the 20 enriched pathways to be directly or indirectly linked to FGF signalling, with the top 5 enriched pathways being greater than 40-fold and specific to FGFR3. The top 2 enriched pathways were linked to the activating mutations of the FGFR3 receptor. As such, subsequent investigation focussed specifically on FGFR3. Interestingly, other enriched pathways included those involved in PI3K/Akt and IGF1R signalling, which have similar overlap with FGFR3 in downstream signalling pathways [26,27].

**Figure 4:**
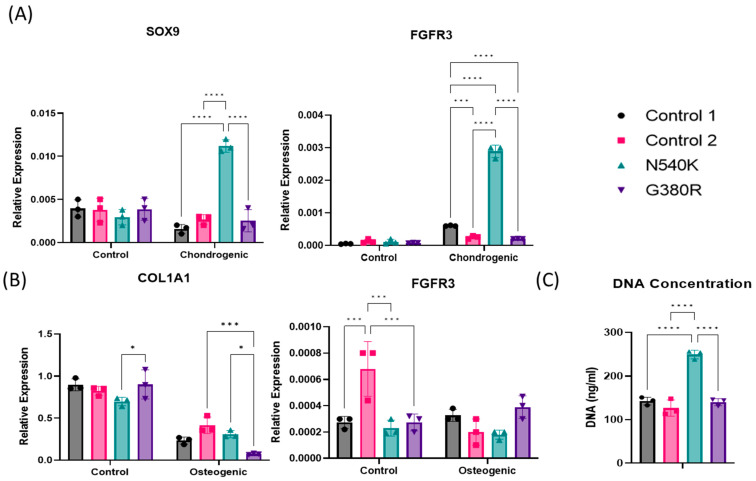
hPDC-FGFR3^N540K^ has enhanced chondrogenic gene expression and proliferative ability in vitro. (**A**) Gene expression analysis of hPDC-FGFR3^N540K^ under chondrogenic conditions shows a 5-fold increase in SOX9 expression and a 7-fold increase in FGFR3 compared to control and FGFR3^G380R^. No differences in SOX9 or FGFR3 expressions were observed under control conditions. (**B**) Gene expression analysis of hPDC-FGFR3^N540K^ under osteogenic conditions displays no increase in expressions of COL1A1 or FGFR3, with a 4-fold reduction in COL1A1 expression observed in FGFR3^G380R^ compared to FGFR3^N540K^ and control. (**C**) Cell quantification via DNA content under control conditions shows hPDC-FGFR3^N540K^ to have a 1.78-fold increase in DNA content compared to control and hPDC-FGFR3^G380R^ (N = 3, * = *p* < 0.05, *** = *p* < 0.001, **** = *p* < 0.0001).

To investigate the effects of activating mutations on hPDC osteochondral differentiation, hPDCs derived from patients harbouring the transmembrane domain 1 activating mutation (N540K) and the extracellular domain mutation (G380R) were cultured under chondrogenic, osteogenic, or control conditions prior to gene expression analysis. As shown in Figure 4, when cultured under chondrogenic conditions, hPDC-FGFR3^N540K^ displays a 5-fold increase in SRY-box transcription factor 9 (SOX9) and a 7-fold increase in FGFR3 expression compared to controls and hPDC-FGFR3^G380R^, with no difference in expression observed under control conditions, suggesting that FGFR3^N540K^ not only enhances chondrogenic gene expression but also acts in a positive feedback manner to promote the expression of FGFR3 itself in vitro. When cultured under osteogenic conditions, there was no significant difference between the hPDC-FGFR3^N540K^ expression of COL1A1 or FGFR3 compared to the control. However, a 4-fold reduction in COL1A1 expression was observed in hPDC-FGFR3^G380R^ compared to the control and hPDC-FGFR3^N540K^. Together, these gene expression data suggest that FGFR3^N540K^ has more potent implications in chondrogenesis with limited effects on osteoblastogenesis in the key genes investigated.

To determine whether the hPDC-FGFR3-activating mutations promote cell proliferation, hPDCs were cultured under standard growth media conditions, and the cell number was inferred from the isolated DNA concentration. Similar DNA concentrations were obtained in the control and hPDC-FGFR3^G380R^ cultures; however, hPDC-FGFR3^N540K^ displayed a 1.78-fold increase in the DNA concentration, which was suggestive of increased cell proliferative capacity.

**Figure 5:**
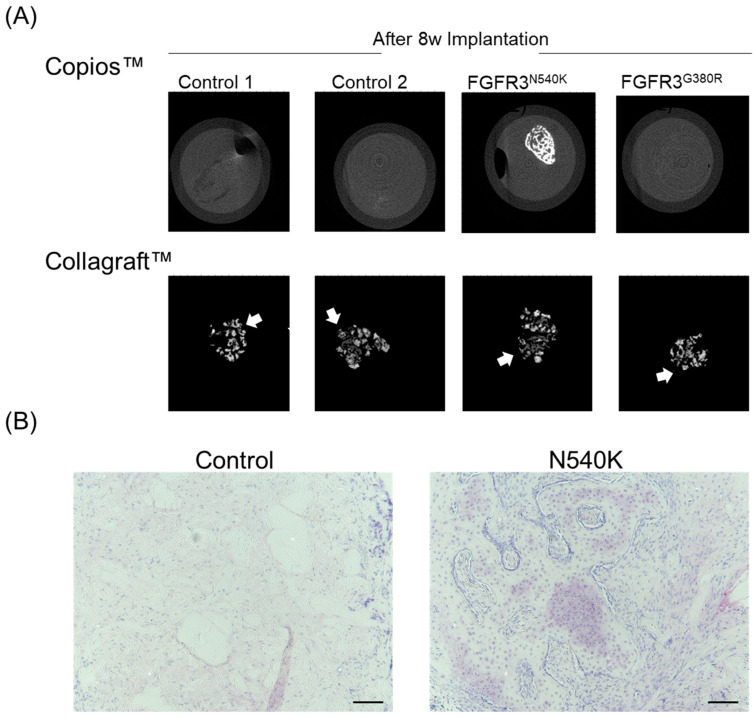
hPDC-FGFR3^N540K^ displays enhanced endochondral bone-forming capabilities in vivo. (**A**) µCT images of hPDC and hPDC-FGFR3(ca) scaffolds following 8-week implantation. Note that bone formation on the Copios™ bone void filler scaffold is only present in hPDC-FGFR3^N540K^. All hPDCs in the Collagraft™ osteoconductive scaffold displayed bone-forming capacity (white arrows). (**B**) Histological analysis of Copios™ scaffold 8 weeks post implantation via Toluidine blue staining shows increased staining in N540K compared to control, with the presence of cartilaginous remnants being indicative of endochondral bone formation (scale bar = 200 µm).

To investigate the implications of constitutively activated FGFR3 mutations in hPDC(ca) osteochondral differentiation in vivo, hPDC-FGFR3^N540K^, hPDC^G380R^, and control hPDCs were seeded onto either Copios™ or Collagraft™ scaffolds (Figure 5). Copios scaffolds are bone void fillers composed of a collagen matrix and have low osteoinductive properties with hPDCs [23], whereas Collagraft scaffolds are collagen–hydroxyapatite hybrid scaffolds and are highly osteoinductive with hPDCs [28]. In the Copios scaffolds, all hPDC-seeded scaffolds displayed bone formation, displaying that they all have the capacity to form bone. (Of note, the high-intensity regions in the scan are hydroxyapatite components of the Collagraft scaffold and do not reflect bone formation from the hPDCs.) Conversely, no bone formation is visible on the µCT images in the controls or the FGFR3^G380R^ hPDC-seeded Copios scaffolds; however, visible bone formation is evident in the FGFR3^N540K^ scaffolds, suggesting that these cells have greater intrinsic capacities to form bone. A further histological analysis of the sections of the FGFR3^N540K^ Copios scaffolds revealed increased Toluidine blue staining in FGFR3^N540K^ compared to the control scaffolds, with evidence of cartilage remnants throughout, which is indicative of endochondral bone formation.

**Figure 6:**
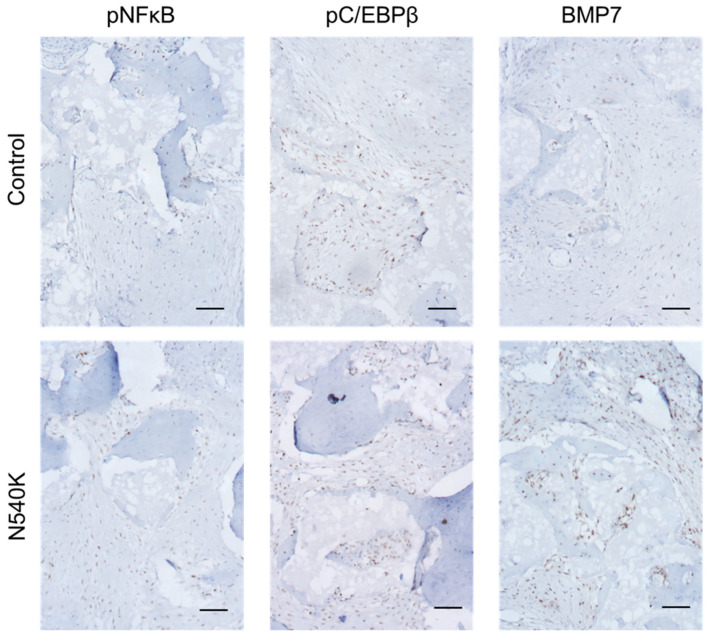
hPDC-FGFR3^N540K^ scaffolds have increased staining for FGFR3-associated targets. (**Left**) An increase in the number of cells positive for phosphorylated NFκB, observed in hPDC-FGFR3^N540K^. (**Middle**) An increase in phosphorylated C/EBPβ, seen in hPDC-FGFR3^N540k^. (**Right**) An increase in BMP7 staining intensity of hPDC-FGFR3^N540K^compared to control (scale bar 200 µm).

Further immunohistochemical analysis of the hPDC-FGFR3^N540K^ Collagraft™-seeded scaffolds was carried out on known downstream targets of FGFR3 signalling (Figure 6), including phospho-NFκB, a downstream target of p38 [29]; phospho-C/EBPβ, a downstream target of ERK1/2 [30]; and BMP7, proposed by Matsushita et al. [31], to be upregulated in chondrocytes expressing activating forms of FGFR3. hPDC-FGFR3^N540K^ containing constructs displayed increased phospho-NFκB with a greater number of phospho-NFκB positive cells throughout compared to the control scaffolds. Additionally, an increase in phospho-C/EBPβ is observed in the hPDC-FGFR3^N540K^ constructs with more widespread positive staining seen throughout the scaffolds and more positive cells observed in comparison to the control. Finally, a notable increase in BMP7 staining is observed in hPDC-FGFR3^N540K^, with minimal staining observed in the control-seeded scaffold, suggesting that hPDC-FGFR3^N540K^ may BMP7 production during endochondral bone formation. Together, these data speculate that the activation of the FGFR3 mutation in hPDCs via N540K may promote enhanced bone formation potentially through p38/ERK- and BMP-related pathways.

## 4. Discussion

It was demonstrated in our previous work that culturing hPDCs in species-matched serum (humanised culture) enhanced their osteochondral potential in vitro [18]; however, despite this striking effect, the processes that were involved remained unknown. Serum composition is complex and differs significantly between batches; we therefore sought to outline an in silico approach to identify novel modulators of osteochondral differentiation in hPDCs. Through the construction of an mRNA-miRNA network, we identified FGFR3 as a key regulator of hPDC osteochondral fate. A pathway analysis implicated that the high-confidence dataset was enriched in genes linked to FGFR3 overactivation mutations. We validated this network with both in vitro and in vivo analyses, showing that FGFR3^N540K^ mutation in hPDCs promotes osteochondral differentiation, resulting in enhanced endochondral ossification, which has not been previously described.

It is clear from this study that the site of the FGFR3 activating mutation determines the effect on the osteochondral differentiation potential of hPDCs, as PRX1^Cre^- and FGFR3^Y637C+^-derived periosteal cells failed to undergo complete endochondral ossification, resulting in pseudoarthrosis and failed fracture repair [32]. These findings align with our data, whereby hPDC-FGFR3^G380R^, another extracellular domain mutant like FGFR3^Y367C^, fails to undergo complete endochondral bone formation, as observed by µCT, in addition to an absence of the induction of any tested endochondral marker in vitro. These extracellular domain mutations likely result in greater signalling bursts akin to ligand-dependent signal induction, which may impede tissue formation, whereby hPDC-FGFR3^N540K^ (tyrosine kinase domain 1 mutation) possibly results in lower-level activation [32]. This may result in altered ERK signalling (a downstream effector of FGFR3 activation), as it is widely documented that the rapid vs. sustained stimulation of ERK determines cell behaviour [30,33,34]. This is further evidenced from the fact that individuals with N540K mutations display less severe forms of achondroplasia, known as hypochondroplasia.

**Figure 7:**
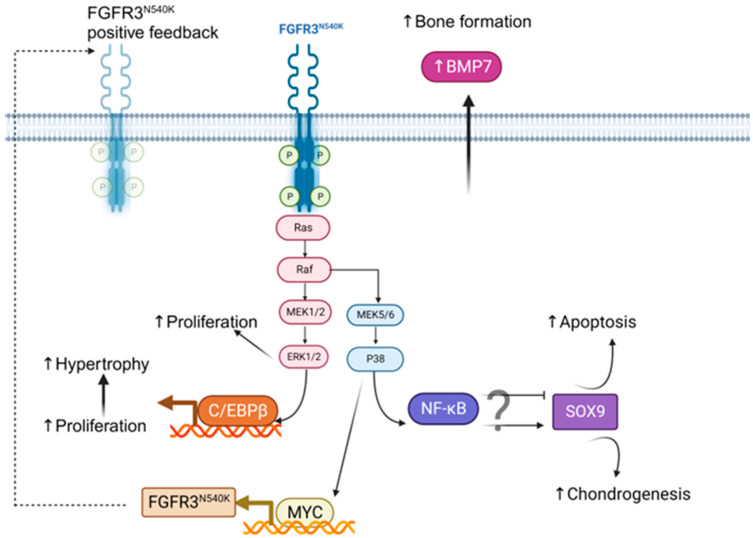
Potential mechanism of hPDC-FGFR3^N540K^-mediated endochondral bone formation in periosteum-derived cells. Kinase domain mutation of N540K permits low-level constitutive activation, which induces mitogenic RAS/ERK pathways. This, in turn, activates p38, resulting in NFκB activation and promoting SOX9 gene expression and subsequent chondrogenesis; additionally, NFκB activation has been linked to chondrocyte apoptosis, which may favour bone formation processes. P38 activation additionally stabilises Myc protein, resulting in accumulation and subsequent increased gene expression of FGFR3^N540K^, resulting in a positive feedback loop ERK1/2 activation directly induces proliferation whilst dampening contact inhibition responses. ERK1/2 also activates C/EBPβ, promoting the switch from chondrocyte proliferation to chondrocyte hypertrophy via SOX9 inhibition, favouring endochondral bone formation. These processes, in addition to those not yet defined, increase BMP7, which functions in a paracrine manner to promote osteoblast-driven bone formation. Created with BioRender.com.

Our in vitro data suggest that the hPDC-FGFR3^N540K^ mutant has a preference for chondrogenic differentiation over osteogenic differentiation with increased endochondral bone-forming capabilities observed on the Copios™ scaffolds. These data conflict the findings by Liosey et al. [35], who report defective bone mineralisation in adult mice harbouring a similar FGFR3 tyrosine kinase domain 1 mutation (N534K). These differences are likely due to the analysis in our studies being cell-specific rather than a global mutation; thus, other cells harbouring the mutation within the bone environment are likely to influence bone dynamics. Further work investigating an hPDC-FGFR3^N540K^ mouse model would provide greater insight to underpin the specific influence of hPDC-FGFR3^N540K^ on bone formation and maintenance. Matsushita et al. [31] identified BMP7 to be upregulated in a mouse model replicating FGFR3^G380R^ in addition to increased bone formation, likely via a paracrine effect on osteoblast differentiation (Figure 7). In our study, hPDC-FGFR3^G380R^ did not display bone-forming capabilities on the Copios™ scaffold as observed with hPDC-FGFR3^N540K^. Although a different mutation (mouse G374 orthologous to human G380R) has been investigated, it is evident that enhanced FGFR3 signalling is related to BMP7 secretion, however the mechanism has not yet been clearly defined. Little is known about the role of BMP signalling in the context of FGFR3^N540K^; however, from our data, it is speculated that this mutation in hPDCs may result in increased BMP7 expression. The crosstalk between the BMP and FGF signalling pathways was reviewed by Schliermann and Nickel [36], where it was elucidated that the FGF and BMPs can function in a synergistic manner to promote chondrocyte hypertrophy via RUNX2 in addition to promoting positive feedback for each signalling cascade. Interestingly, the Erk pathway is important in both signalling cascades [36]. Furthermore, BMP-positive feedback loops have been proposed in Drosophilia and Chordates [37,38]; thus, in the context of our study, it may be speculated that the constitutive kinase domain activation in FGFR3^N540K^ hPDCs promotes BMP signalling via this synergistic interaction, which is further amplified via a positive feedback mechanism.

The activating mutations of FGFR3 have been shown to promote a positive feedback in FGFR3 expression through the stabilisation of Myc (Myc proto-oncogene protein), resulting in the upregulation of FGFR3 in bladder cancers [39]. This is also supported in our dataset, whereby hPDC-FGFR3^N540K^ results in upregulated FGFR3 gene expression under chondrogenic conditions, suggesting that this positive feedback loop may also be present in other cellular contexts.

Our data suggest that hPDC-FGFR3^N540K^ has a greater proliferative capacity compared to FGFR3^G380R^ and the control hPDCs, with an increased DNA content observed in vitro. ERK signalling is activated in response to FGFR3 activation and it is a known mitogenic pathway that promotes cell proliferation [40]. Further evidence for increased ERK signalling is seen in widespread pC/EBPβ staining in the hPDC-FGFR3^N540K^-seeded scaffolds. C/EBPβ alone has also been directly linked to cell proliferation via promoting the expression of mitogenic genes, suggesting that FGFR3^N540K^ cells may lose contact inhibition abilities, resulting in overgrowth [41,42]. Furthermore, C/EBPβ has been shown to promote the transition from proliferative chondrocyte to hypertrophy via the repression of SOX9 activity, thus favouring endochondral bone formation [43]. In our data, pNFκB, a downstream effector of p38, is suggested to be increased in hPDC-FGFR3^N540K^ scaffolds. It has been documented that mitogenic signalling via p38 is required for chondrocyte hypertrophic differentiation [44]. Indeed, NFκB has been shown to positively regulate SOX9 expression with the NFκB binding sites found within the SOX9 promoter in cancer stem cells [45]. Furthermore, NFκB has also been proposed to positively regulate SOX9 activity via a BMP2 axis in chondrocytes [46]. However, during joint inflammation, as seen in OA, NFκB has been linked to the suppression of SOX9 in chondrocytes, favouring cartilage degradation and chondrocyte apoptosis, and likely favouring bone formation processes [47]. Together, these highlight the complex role of NFκB, which is likely cell- and context-dependent.

FGFR3 has been the subject of recent focus in OA research, whereby pathological endochondral ossification processes are observed within the articular cartilage. Patients carrying FGFR3-activating mutations are thought to be protected from OA development, with FGFR3 activation exerting a cartilage protective effect via the inhibition of chondrocyte hypertrophy, a key process in endochondral ossification [48,49,50]. Our data suggest that the effects that FGFR3 activation has on endochondral ossification is cell-/location-specific, with an overactivation in hPDCs promoting endochondral ossification. During OA development, chondrocytes within the articular cartilage switch from a stable state, maintaining cartilage integrity, to hypertrophy akin to the differentiation processes observed during endochondral ossification [51]. Additionally, endochondral processes are observed during osteophyte formation in OA, which originate from the periosteum and are thought to be a defence mechanism against joint instability [13,52]. Interestingly, the data by Moore et al. suggest that FGF18 (FGFR3 ligand) treatment in an OA rat model promotes “chondrophyte” formation [53], a term described by the research group as “early osteophyte formations”, which, if given more time, would likely form osteophytes via endochondral ossification. Furthermore, FGF18 treatment, known as Sprifermin, has been further developed as a potential OA therapeutic option, which has displayed mixed results in clinical trials. Sprifermin failed to pass phase II trials due to having no positive effect on symptom alleviation, and treatment had no effect on osteophyte formation after 24 months [54]. A 5-year follow-up of the FORWARD study did, however, suggest that Sprifermin treatment offered long-term structural joint modification with clinically relevant pain reduction, although reports on osteophyte formation were not detailed [55]. It should be noted that modifying osteophyte growth pharmacologically would likely not be desirable due to them being formed in response to an altered loading, which is a tightly controlled process.

In our dataset, mir-145 was the top downregulated DEMi and has implications in cartilage and bone biology. Mir-145 expression has been linked to the inhibition of osteogenesis in the human osteoblast cell line hFOB in vitro, with suppression enhancing osteogenic differentiation in these cells via the modulation of semaphorin 3A [56]. Furthermore, mir-145 has been found to be a direct regulator of SRY-box transcription factor 9 (SOX9), the master regulator of chondrogenesis, in addition to other genes involved in chondrocyte biology [57,58,59]; this aligns with our data, as SOX9 gene expression is increased in FGFR3^N540K^. Mir-Let-7 was found to be the second downregulated miRNA in our dataset and has also been implicated in osteoblast mineralisation, with its inhibition found to enhance periodontal ligament stem cell osteogenic differentiation [60] in addition to modulating chondrocyte differentiation in the growth plate. Interestingly, mice lacking a combination of functional mir-Let-7 and mir-140 displayed severe skeletal growth defects, implicating a synergistic role of these miRNAs in chondrocyte differentiation and overall skeletal development [61]. MiR-106a-5p was identified as one of the top upregulated DEMis in our dataset and has been implicated as a potential therapeutic target for OA. miR-106a-5p expression is downregulated in patient tissue, and intra-articular injection of an miR-106a-5p agomir alleviated the disease phenotype in an ACLT mouse model of OA [62]. Furthermore, miR-106a-5p has been shown to directly target BMP2 in mesenchymal stromal cells, thereby inhibiting the TGFβ/BMP signalling pathway, which has significant implications in fracture healing [63]. Taken together, these data suggest that the DEMis in our dataset have prominent roles in osteochondral differentiation, providing further validation of the miRNA-mRNA network that was generated.

## 5. Conclusions

In conclusion, we believe that we have generated a high-confidence mRNA-miRNA network, which identified FGFR3 as a key network node, which was validated in vitro and in vivo. The platforms of in silico and functional analyses permit the identification of further modulators of hPDC osteochondral differentiation and reveal the potential future for miRNAs or their inhibitors as potential novel therapeutic strategies to control aberrant osteochondral differentiation present in fracture non-union and OA.

## Data Availability

The data presented in this study are available upon request from the corresponding authors.
